# Particle flow numerical simulation model for coal and gas outburst and its microscopic outburst mechanism description

**DOI:** 10.1038/s41598-024-52973-w

**Published:** 2024-02-03

**Authors:** Huice Jiao, Weihua Song

**Affiliations:** 1 State Key Laboratory of Coking Coal Exploitation and Comprehensive Utilization, China Pingmei Shenma Corporation Group, Pingdingshan, 467000 China; 2 College of Mining, Liaoning Technical University, Fuxin, 123000 China

**Keywords:** Energy science and technology, Engineering

## Abstract

The finite difference model and particle flow model were constructed to reproduce the process of coal and gas outburst in front of the tunnel. The reliability of the particle flow model was verified by studying the internal stress, gas pressure, and related parameters of gas and coal two-phase flow. Then the process of coal and gas outburst is described from a microscopic perspective. The results show that the particle flow numerical model of coal and gas outburst is in line with reality and can intuitively and conveniently monitor relevant parameters; The concentrated stress caused by excavation of tunnels can reach 1.2 times the in-situ stress; High stress causes the adhesion between coal particles to break down, which is the main reason for the initiation of coal and gas outbursts; The intermittent breakthrough of gas on the coal particle collection that blocks the release channel has caused pulsating characteristics of coal and gas outbursts.

## Introduction

Coal and gas outburst refers to the rapid movement of gas containing coal and rock masses underground in coal mines towards the excavation space in a crushed state, accompanied by a violent dynamic process of a large amount of gas emission, which seriously threatens the safe production of coal mines ^1,2^. The early hypotheses were single factor hypotheses ^3,4^, such as gas effect hypothesis, crustal stress effect hypothesis, chemical essence hypothesis, and comprehensive action hypothesis, such as the energy hypothesis ^5^. However, due to the complexity of the coal and gas outburst process and the numerous contributing factors, the understanding of the causes, processes, and some details of the outburst occurrence is not yet clear. In recent years, Chinese scholars have conducted extensive research on the mechanism of coal and gas outburst through theoretical analysis, physical simulation, and numerical simulation experiments, and proposed a series of theories such as the central expansion theory ^6^, the two-phase fluid hypothesis ^7^, the rheological hypothesis ^8^, and the spherical instability hypothesis ^9^, which have greatly enriched and improved the basic theory of coal and gas outburst.

With the rapid development of computer technology, numerical simulation has been widely applied in the study of coal and gas outburst due to its advantages of short research cycle, adjustable simulation parameters, and independence from experimental environment. Yu et al. ^10^ used 3DEC numerical simulation software to study the relationship between fault angle and roof pressure, and then analyzed the influence of fault angle on coal and gas outburst. Wang et al. ^11^ analyzed the fracture development of coal and surrounding rock in the blasting mining face and the impact of blasting disturbance on the adsorption capacity of gas using theoretical calculations and UDEC numerical simulation software, thereby obtaining the mechanism of blasting disturbance-induced coal and gas outburst. Zhu et al. ^12^ used RFPA^2D^-Flow numerical calculation software to numerically study the occurrence and development process of coal and gas outbursts under different ground stresses and gas pressure conditions. They obtained the laws of crack initiation, propagation and evolution in coal bodies, as well as the characteristics of outburst pore changes. Rong et al. ^13^ used FLAC^3D^ and COMSOL numerical simulation methods to analyze the impact of excavation on coal and gas outbursts, in order to optimize the mine’s risk mitigation plan. Cao et al. ^14^ simulated the stress field, deformation field, and gas flow field distribution of red coal during coal and gas outbursts using COMSOL software based on the gas–solid coupling equation of gas containing coal. Zheng et al. ^15^ used the FLAC^3D^ numerical simulation method to analyze the mechanism of strong and weak coupling structures for preventing coal and gas outbursts from an energy perspective. Li et al. ^16^ used the COMSOL numerical simulation software to establish a two-dimensional mine geological model containing fault structures, and studied the distribution of ground stress, gas pressure, and gas migration laws of coal seam working faces at different distances from the fault during mining operations.Wang et al. ^17^ conducted numerical simulations of coal and gas outbursts using the PFC^3D^ simulation software, reproducing the accumulation process of coal bodies. Tang et al. ^18^ used PFC^3D^ and FLAC^3D^ programs as a platform to verify the validity of the effective stress theory formula for different zones in front of the coal mining face using numerical simulation methods.

Due to the complexity of the coal and gas outburst process, there are still differences between theoretical hypotheses, physical simulation, numerical simulation, and reality.The particle flow numerical simulation method is intuitive for simulating the outburst process, and it is also convenient for studying the changes in stress in the coal body, the flow rules of gas, the expansion mode of coal cracks, and the velocity of coal near the outburst outlet. Therefore, the use of particle flow numerical simulation method can serve as a bridge between the macroscopic phenomenon and theory of coal and gas outburst.In this paper, the FLAC^3D^ numerical simulation software is used to analyze the stress distribution state in front of the roadway, and the PFC^3D^ particle flow numerical simulation software and CFD fluid module are used to simulate the process of coal and gas outburst, verify the general law of coal and gas outburst, and analyze the occurrence mechanism of coal and gas outburst from a microscopic perspective.

## Redistribution law of stress in front of excavation face

The occurrence of coal and gas outbursts is closely related to the stress field, and high stress is an important factor in inducing coal and gas outbursts^19^. The tunneling face is a high-risk area for coal and gas outbursts, and it is necessary to study the characteristics of the stress ahead of the tunneling activity on the working face ^20,21^.

**Figure 1:**
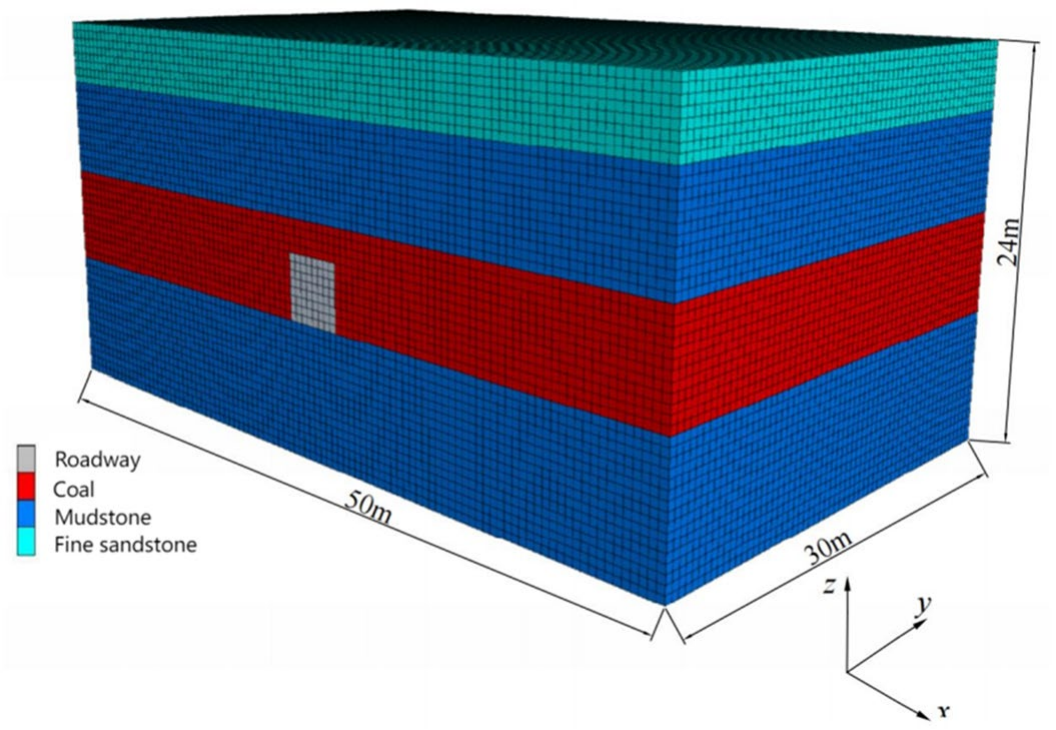
The three-dimensional diagram of the model.

A FLAC^3D^ numerical simulation model was established based on the geological information of a certain mine in Pingdingshan mining area. The model is 50 m long, 30 m wide, and 24 m high, as shown in Fig. 1. The model parameters are shown in Table 1, and the boundary conditions of the model are taken from reference ^22^. Among them, the x direction is the minimum horizontal principal stress direction, the y direction is the maximum horizontal principal stress direction, and the z direction is the vertical stress direction, which are 11.82 MPa, 16.71 MPa, and 13.35 MPa, respectively.

**Table 1:** Stratigraphic characteristics and coal rock mechanical parameters table.

No.	Rock formation	Thickness/(m)	Density/(kg m^−3^)	Bulk modulus/(GPa)	Shear modulus/(GPa)	Cohesion/(MPa)	Internal friction angle/(°)
4	Fine sandstone	4	2667	7.5	8.0	12	53
3	Mudstone	6	2410	4.9	3.2	70	50
2	Coal	6	1330	0.33	0.14	1.71	34.8
1	Mudstone	8	2410	4.9	3.2	70	50

**Figure 2:**
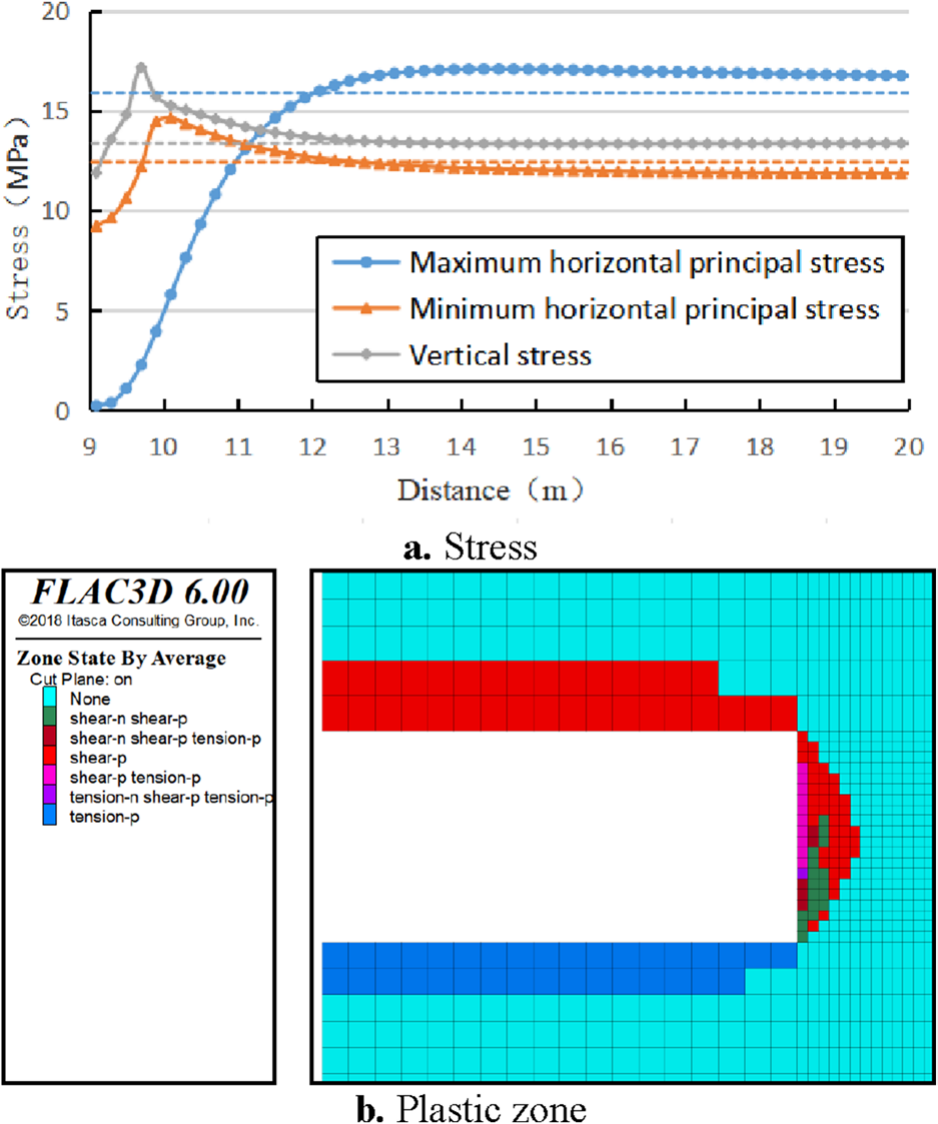
Distribution of stress and plastic zone in front of excavation face.

The stress redistribution characteristics and plastic zone distribution in front of the excavation face are shown in Fig. 2. In front of the excavation face, there is a stress increase zone in both the minimum horizontal principal stress and the vertical stress direction, while there is no significant stress increase zone in the maximum horizontal principal stress direction (radial direction of the working face). The limit equilibrium zone in front of the excavation face ranges from 0 to 0.7 m, the pressure relief zone ranges from 0 to 0.3 m, the stress increase zone ranges from 0.3 to 2.1 m, and the depth of the plastic zone is about 1.2 m. When not excavated, the vertical stress is slightly less than the maximum horizontal principal stress. But after the excavation of the tunnel, the vertical stress reaches its maximum value at 0.7 m in front of the working face, with a maximum value of 17.14 MPa, which is 1.28 times the vertical in-situ stress; The minimum horizontal principal stress reaches its maximum value near 0.9 m in front of the working face, which is 14.62 MPa, which is 1.23 times the in-situ stress; The maximum horizontal principal stress is relieved at the coal wall of the working face and restored to the original stress at a distance of 2.9 m from the working face.

## Establishment of particle flow model

### Calibration of coal micro parameters

Brazilian splitting and uniaxial compression tests were conducted on the collected coal samples in the laboratory, and the test results are shown in Table 2. Then, the particle flow numerical simulation models for uniaxial compression tests and Brazilian splitting tests were established, and the model size was consistent with the laboratory specimen size. The average radius of the particles in the model is 2 mm, and the ratio of the maximum to minimum radius of the particles is 1.66. The model adopts the Parallel Bond Model (PBM), and the particle flow model predicts the microscopic parameters as shown in Table 3.

**Table 2:** Results of laboratory tests.

Number	Size/(mm)			Maximum load/(kN)	Compressive strength or tensile strength/(MPa)	Average compressive strength/(MPa)
	Length	Width	Height			
MKY-1	51.03	51.63	100.42	14.51	5.51	5.29
MKY-2	50.77	50.43	100.36	13.70	5.35	
MKY-3	49.40	49.79	99.74	12.31	5.00	
MKL-1*	50.61	–	24.27	0.99	0.51	0.49
MKL-2*	50.46	–	24.76	1.37	0.70	
MKL-3*	50.19	–	25.88	0.56	0.27	

Note: * means the specimen for the Brazilian splitting test, which is circular in shape and the size parameters are diameter and height.

**Table 3:** Microscopic parameters for particle flow simulation.

Radius of the smallest particle (R_min)_/(mm)	Ratio of the radius of the largest and smallest particles (R_max/_R_min)_	Effective modulus (E_c_)/(GPa)	Ratio of normal to tangential stiffness of particles (K_n_/K_s)_	Frictional coefficient
1.2	1.66	3 × 10	2	0.577
Effective modulus of parallel bonding ($\overline{E}$)/(GPa)	Ratio of normal to tangential stiffness of parallel bonding (${{\overline{{K_{n} }} } \mathord{\left/ {\vphantom {{\overline{{K_{n} }} } {\overline{{K_{s} }} }}} \right. \kern-0pt} {\overline{{K_{s} }} }}$)	Tensile strength/(MPa)	Cohesion/(MPa)	Normal contact damping
3 × 10	2	5.29 × 10	1.8 × 10	0.5

**Figure 3:**
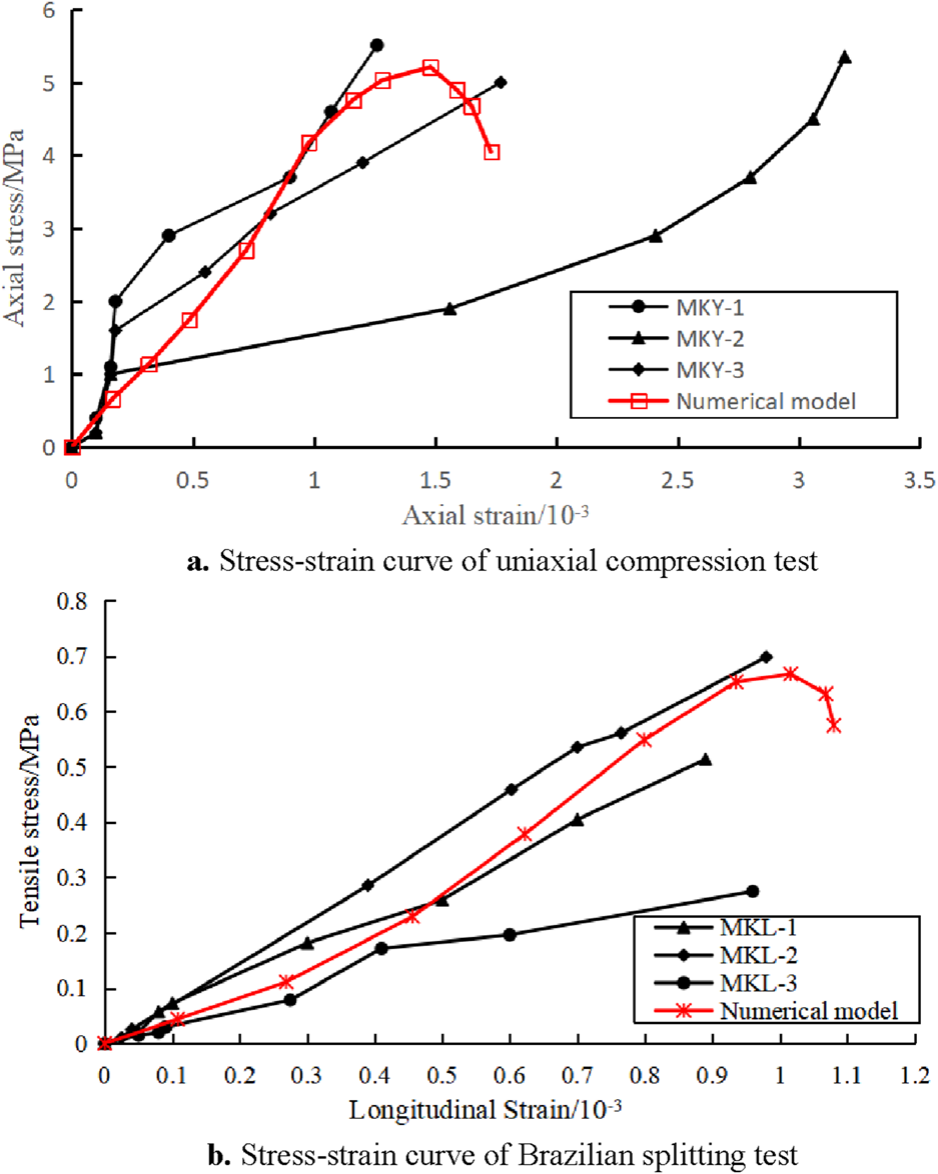
Comparison between laboratory test results and simulation test results.

The comparison diagram of laboratory test and numerical simulation test results is shown in Fig. 3. The average values of the elastic modulus, uniaxial compressive strength, and uniaxial tensile strength of coal obtained from laboratory tests are 0.37 MPa, 5.29 MPa, and 0.49 MPa, respectively. The data obtained from PFC simulation tests are 0.37 MPa, 5.21 MPa, and 0.57 MPa, respectively, with deviations of 0, 1.51%, and 16.3%. The microscopic parameters of the particle flow model basically meet the requirements.

### Model establishment

**Figure 4:**
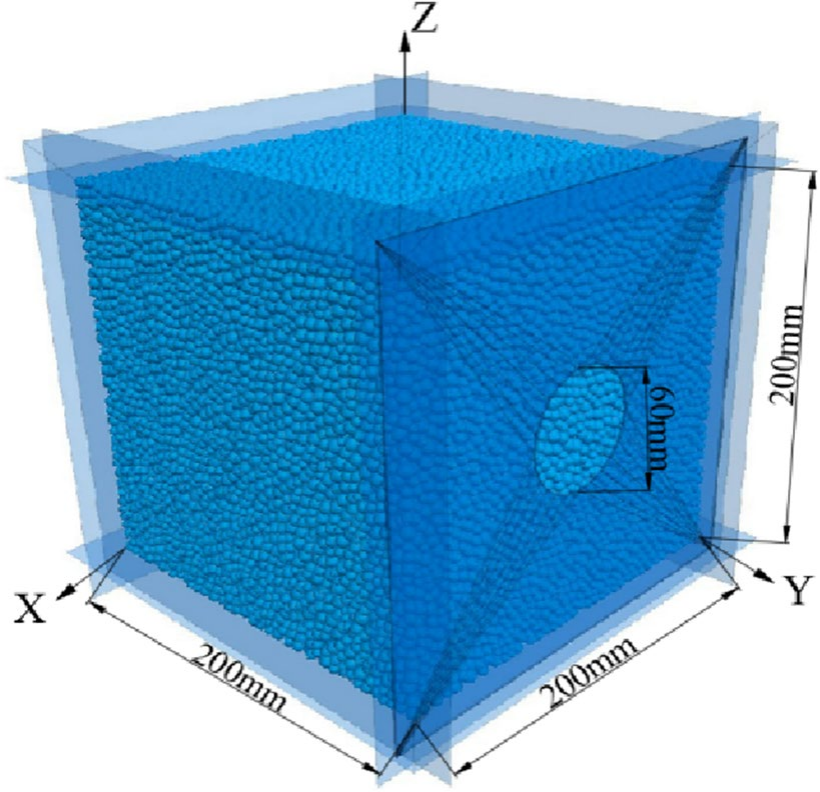
Schematic diagram of coal and gas outburst model.

The model size is 200 mm × 200 mm × 200 mm (Fig. 4). The diameter of the coal and gas outburst window is 60 mm, and the coal particle diameter ranges from 2 to 3.4 mm, with a particle count of 27,272. Based on the model size and particle diameter, the fluid grid edge length was set to 8 mm, and a total of 15,625 grids were divided.

### Fluid parameters

The parameters of the fluid mainly include fluid reference density, dynamic viscosity, fluid reference pressure, fluid compressibility coefficient, and other parameters. The specific values refer to reference ^23^, as shown in Table 4.

**Table 4:** Table of fluid parameters.

Density/(kg m^−3^)	1.97
Dynamic viscosity/(N s m^−2^)	1.5 × 10^−5^
Fluid compressibility coefficient	2.0 × 10^−5^

### Principle of fluid structure coupling

The compute fluid dynamics (CFD) module built-in in PFC is used to construct a fluid structure coupling model to describe the process of coal and gas outburst, and the coarse grid method is used to solve the interaction problem between fluid and particles. In the coarse grid method, the motion equation of the fluid is numerically solved on a set of fluid elements larger than PFC particles, and the force of the fluid on the particles is distributed to local particles based on the fluid conditions. The corresponding physical force acts on the fluid in the form of an average, and porosity and fluid drag are calculated by the average parameters of the particles acting on the fluid unit. The bidirectional coupling between particles and fluid is achieved through the periodic exchange of this information between PFC and fluid flow solver.

The process of coal and gas outburst is extremely complex, and its mechanism is not yet fully understood. Therefore, it is necessary to simplify the process and make the following assumptions for the particle flow fluid structure coupling model: ① Positive pressure fluid: fluid density ρ is related to pressure P and independent of temperature. ② In the process of coal and gas outburst, the flow of gas in the coal body can be regarded as low Reynolds number flow, which can be described by the Darcy's law. ③ The adsorption and desorption effects of gas during coal and gas outbursts are not considered, and the gas compression coefficient is small enough to be negligible. ④ The flow speed of gas is slower than that of sound.

### Simulation scheme design

The process of coal and gas outburst is extremely complex, and it is difficult to fully simulate the entire outburst process using particle flow numerical simulation methods. The initial stage of prominence can characterize the intensity and main characteristics of prominence, so this stage can be considered as the focus of research. At this stage, it can be assumed that the gas flowing in the coal body can be continuously replenished by the gas released from the rear coal body, that is, during the process of coal and gas outburst, the gas continuously enters along the Y-axis direction with stable pressure, and the flow of fluid in the coal body conforms to Darcy’s law.

In order to reflect the characteristics of coal and gas outbursts in practice, it is necessary to determine the similarity ratio between the model and actual engineering. According to references ^24^, the stress similarity constant $C_{\sigma }$ is equal to the geometric similarity constant $C_{L}$, that is

1 $$ C_{\sigma } = C_{L} = {{L_{p} } \mathord{\left/ {\vphantom {{L_{p} } {L_{m} }}} \right. \kern-0pt} {L_{m} }} = \sqrt {{{A_{p} } \mathord{\left/ {\vphantom {{A_{p} } {A_{m} }}} \right. \kern-0pt} {A_{m} }}} $$

In the formula: $L_{p}$ is the size of the prototype; $L_{m}$ is the size of the model; $A_{p}$ is the area of the prototype; $A_{m}$ is the area of the model. The area of the simulated excavation face roadway is approximately 15 m^2^, and the area of the coal and gas outburst plane in the particle flow model is 0.04 m^2^. After calculation, the stress similarity constant $C_{\sigma }$ is 19.36.

Based on the stress redistribution law of the coal body in front of the excavation face obtained from the previous research, the three-dimensional stress data of the plane where the maximum stress is located and the gas occurrence conditions at that elevation are selected. Design the stress and gas pressure for numerical simulation, with a stress similarity constant of $C_{\sigma } = 19.36$ and a gas pressure similarity constant of $C_{p} = 1:1$, as shown in Table 5.

**Table 5:** Table of numerical simulation test scheme.

Stress/(MPa)			Gas pressure/(MPa)
X	Y	Z	
0.63	0.86	0.89	1.41

### Measuring ball settings

**Figure 5:**
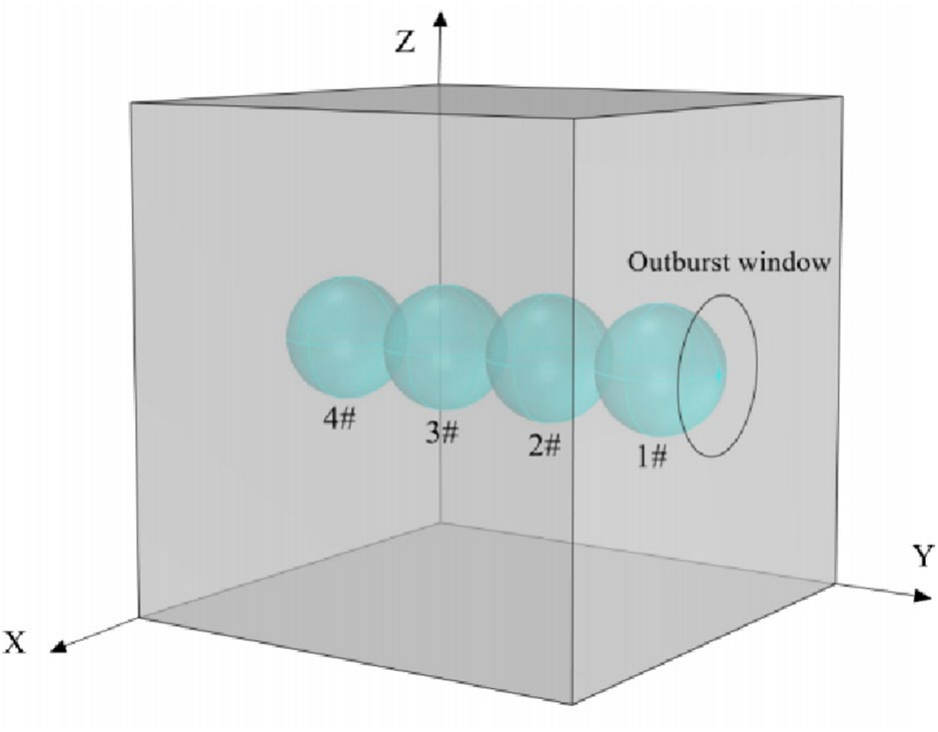
Schematic diagram of measuring ball position.

Set four measuring balls along the centerline of the outburst window with a radius of 25 mm. The numbering and relative position are shown in Fig. 5.

## Numerical simulation analysis of particle flow in coal and gas outburst

### Analysis of the characteristics of coal and gas outburst areas

The force chain is a bridge between micro particle systems and macro systems, and through the transfer process of forces between particles, it can characterize the development process of coal and gas outbursts.

**Figure 6:**
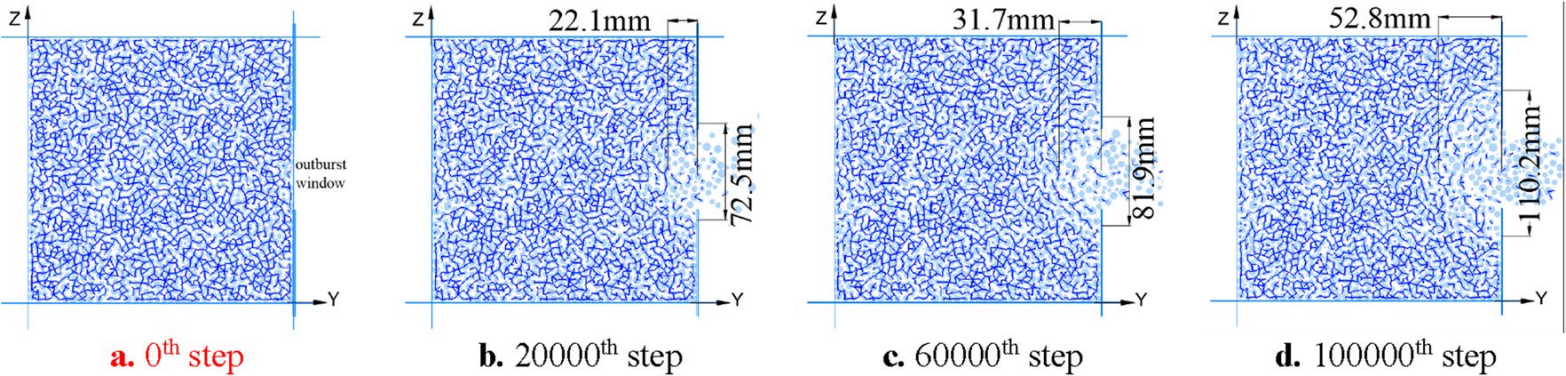
Evolution of force chain in middling coal during coal and gas outburst.

The variation process of the force chain between particles on the central plane of the outburst window (x = 100 mm) is shown in Fig. 6. Figure 6a shows the initial state of the coal body, where the internal force chain is uniformly distributed and the integrity of the coal body is good; When loaded to 20,000 steps (Fig. 6b), the coal near the outburst window was first destroyed, forming a force chain fracture area with a depth of 22.1 mm and a height of 72.5 mm, with an area of approximately 2.52 × 10^3^ mm^2^; When loaded to 60,000 steps (Fig. 6c), the force chain continued to be broken and extended deeper, forming a force chain fracture area of 31.7 mm deep and 81.9 mm high in the coal body, with an area of approximately 4.08 × 10^3^ mm^2^; When loaded to 100,000 steps (Fig. 6d), the damage area further expands, and the interaction force between coal particles weakens, forming a force chain fracture area with a depth of 52.8 mm and a height of 110.2 mm, with an area of approximately 9.13 × 10^3^ mm^2^.

### Evolution law of stress inside coal body

**Figure 7:**
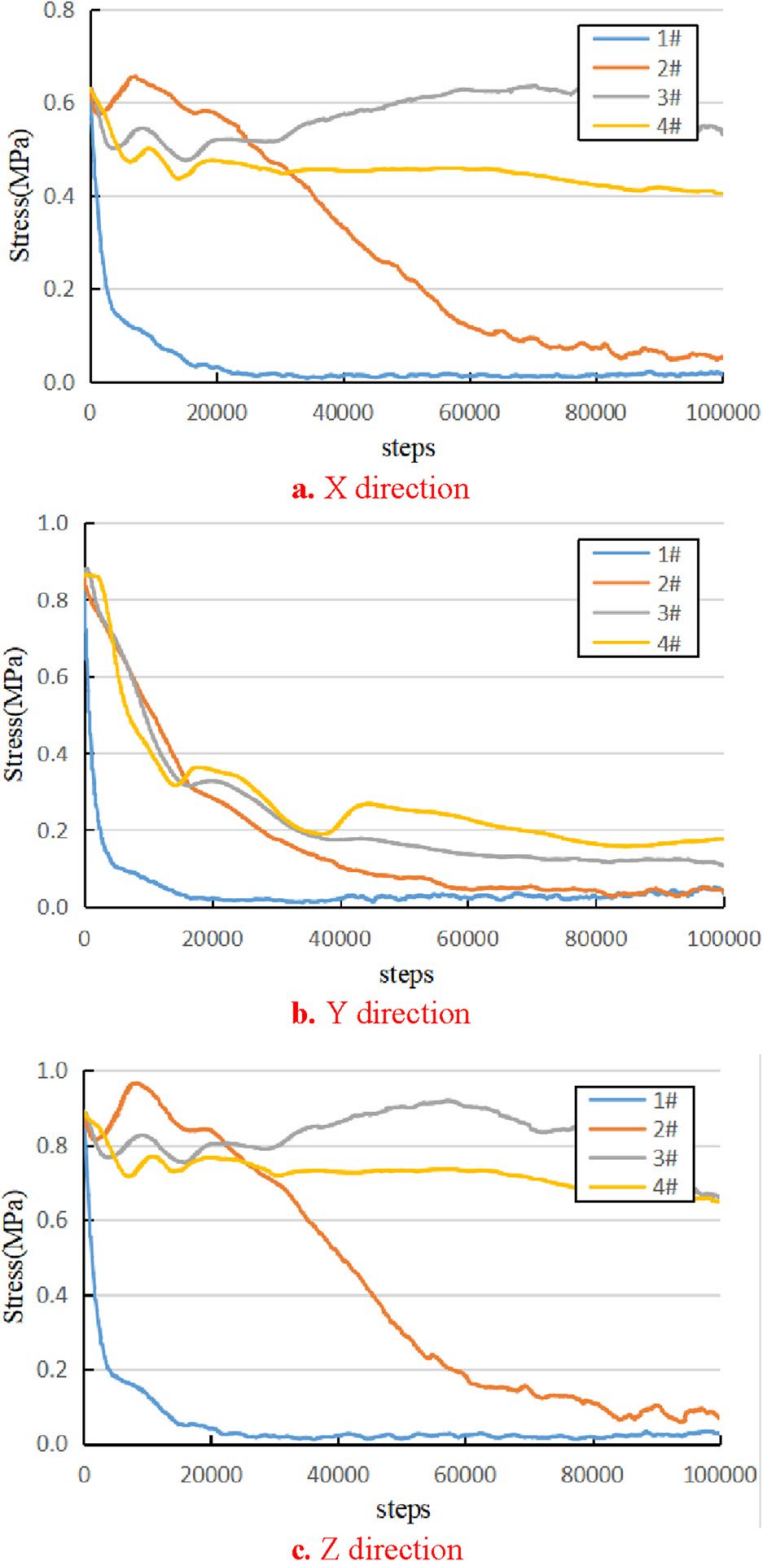
Evolution of internal stress in coal body model.

The process of coal and gas outburst is a continuous transfer of stress within the coal body. The stress in different areas of the coal body is monitored by measurement balls. Figure 7a and c show the stress evolution curves in the X and Z directions perpendicular to the outburst window, respectively, and their variation patterns are similar. After the outburst window is opened, the stress state of the coal body near the 1 # measuring ball changes from three directions to two directions. The elastic energy and gas energy accumulated in the coal body are rapidly released outward, causing the coal body to immediately become unstable and damaged, and the stress inside the coal body also rapidly decreases. With the development of coal and gas outbursts, the stress of the 2 # measuring ball gradually decreases after a small increase; the stress of measurement balls 3 # and 4 # experienced a small fluctuation and slight decrease within 100,000 steps, but their amplitude of change was much smaller than the 1 # and 2 # measurement balls closer to the outburst window. It can be seen that as coal and gas outbursts develop deeper, expanding cavities appear inside the coal body, and the stress inside the coal body also decreases continuously. Figure 7b shows the stress changes in the Y-axis direction inside the coal body, which is the direction of the coal and gas outburst channel. In this direction, the internal stress of the coal body has a similar trend to the stress in the x and z directions, but its decreasing speed is faster, and the stress of the four measuring balls decreases rapidly from near to far.

### Flow law of gas

**Figure 8:**
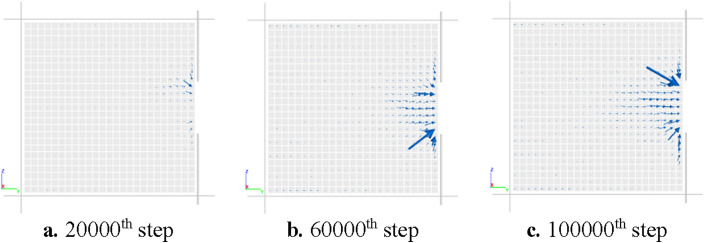
Evolution diagram of gas flow field during outburst process.

The flow field diagrams of gas migration on the x = 100 mm plane in steps 20,000, 60,000, and 100,000 after the outburst window is opened were drawn, as shown in Fig. 8. The arrow represents the direction of gas flow, while the size of the arrow represents the magnitude of gas flow velocity. It can be seen that at 20,000 steps, the flow rate of gas near the outburst window is much higher than that in the deep coal body. Especially, the flow rate of gas around the outburst window is higher than that in the Y direction; At 60,000 steps, due to the expansion of pores in the coal body near the outburst window, gas flows out of the hole in an approximately symmetrical arc at the center of the hole, and the area of rapid gas flow in the coal body also expands; At 100,000 steps, the coal and gas outburst continued to expand deeper, and a large number of cracks appeared in the coal body. The generation of cracks provided a channel for the migration of gas in the coal body, leading to a continued increase in gas flow rate.

### Characteristics of coal particle movement

**Figure 9:**
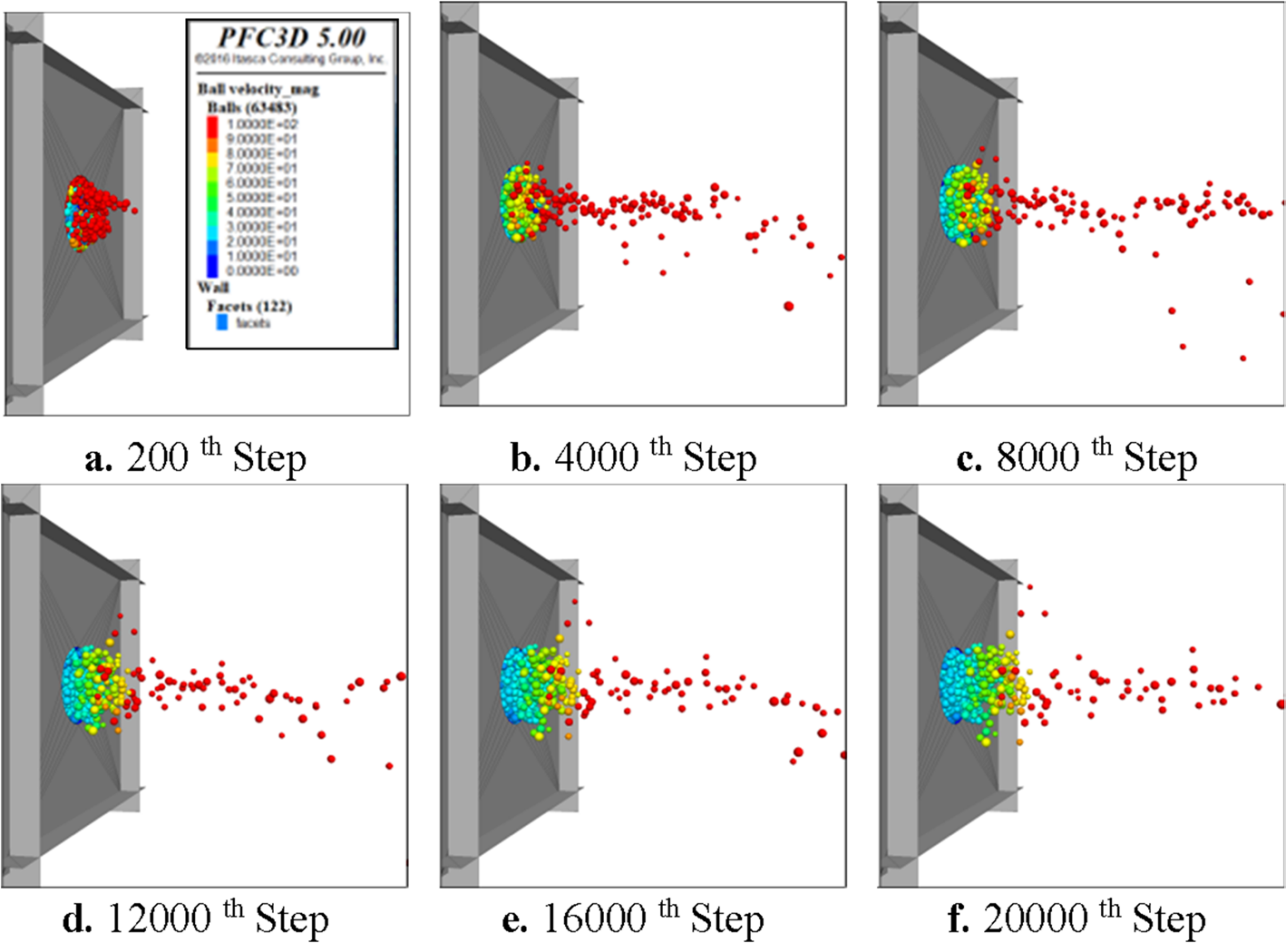
The movement characteristics of coal particles.

**Figure 10:**
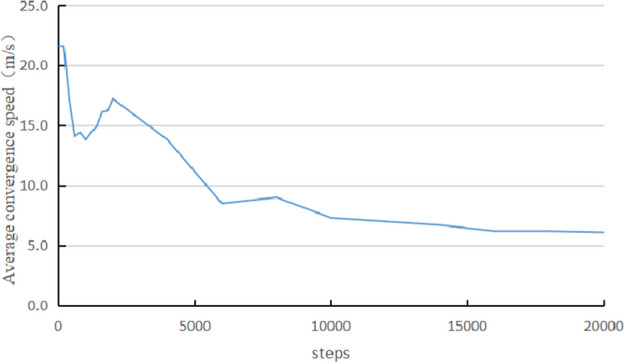
Evolution curve of combined velocity of particles.

Coal and gas outburst is an extremely intense and brief process. Studying the velocity of coal particles passing through the outburst window has important reference value for the scale of energy released at the moment of coal and gas outburst occurrence. Figures 9 and 10 show the particle motion characteristics and the average combined velocity evolution of particles passing through the outburst window. After the start of coal and gas outburst, some coal particles are immediately ejected outward, and the average combined velocity of the particles is about 21.6 m s^−1^; subsequently, the velocity of particles passing through the outburst window rapidly decreases. But at step 1200, the average particle velocity gradually increases and then decreases again after reaching 17.2 m s^−1^ (step 2000). In step 6000, the average velocity of particles fluctuated slightly and continued to decrease. From Fig. 10b–d, it can be seen that a large number of coal particles are ejected at this time, but the particle velocity significantly decreases.

It can be seen that the coal and gas outburst process simulated by the particle flow model also has obvious staged and pulsating characteristics. The internal stress and gas release process of the coal body are consistent with the physical simulation test results of coal and gas outburst ^25^. Therefore, the reliability of this particle flow model can be confirmed.

## Microscopic description of coal and gas outburst mechanisms

**Figure 11:**
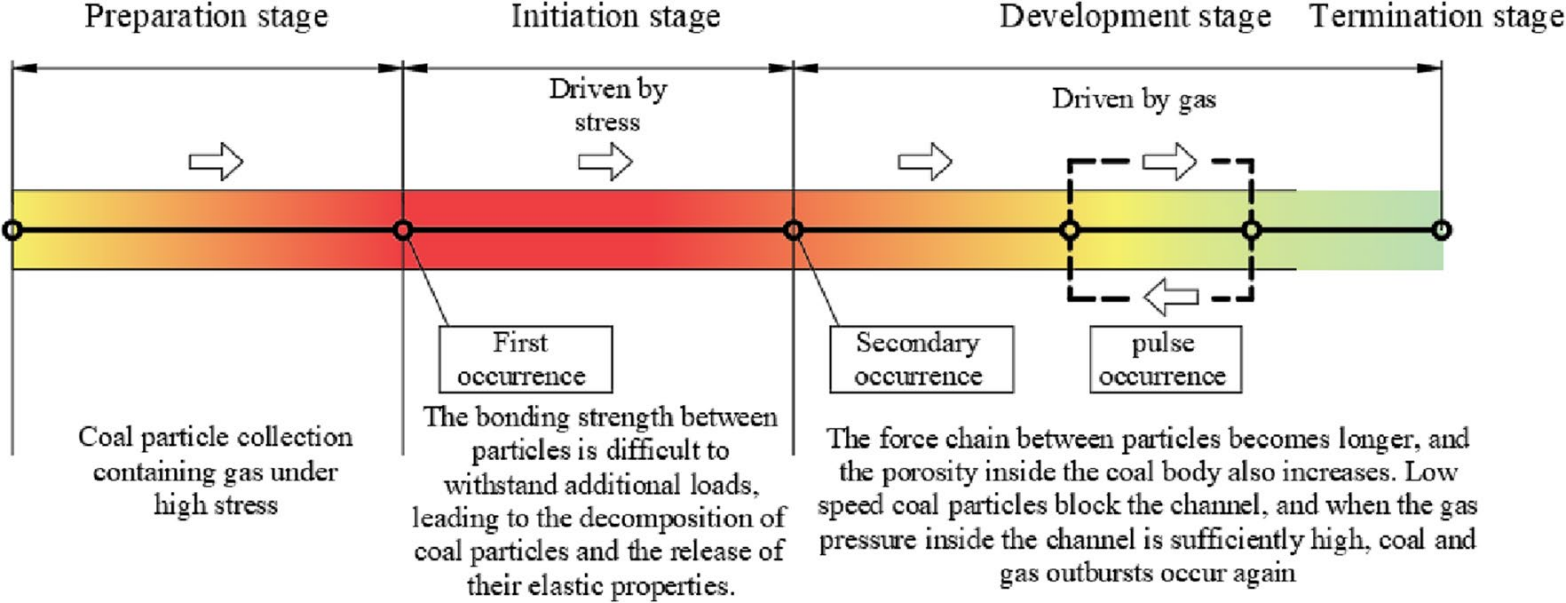
Microscopic description diagram of coal and gas outburst process.

Based on the results of particle flow numerical simulation and previous research by scholars ^26,27^, the process of coal and gas outburst described from a microscopic perspective is shown in Fig. 11.

1. Preparation stage.

When excavating in a fractured and gas rich coal body, the bonded coal particles in front of the working face are compressed under the combined effect of in-situ stress and concentrated stress caused by excavation activities. The force chain between coal particles is shortened and elastic properties are accumulated, and the coal body enters the preparation stage. Coal in the preparation stage is commonly present.

2. Initiation stage.

The initiation stage is a sudden change stage in the initiation of coal and gas outbursts, which has considerable complexity and randomness. When a large number of bonded coal particles reach the critical state of bonding and fragmentation, external disturbance events such as fault stacking, abnormal changes in coal seam thickness, and strong mining disturbances (such as blasting) also occur. The bonding strength between particles is difficult to withstand additional loads, leading to the disintegration of coal particles. At this point, the elastic energy of the coal body is violently released to form shock waves, and a small amount of coal particles are also thrown out at high speed, causing damage to the roadway and facilities. In addition, during this stage, the coal particles are relatively dense, and the gas adsorbed on the coal particles does not have enough space for large-scale desorption, resulting in a slow gas flow rate in the coal body. Therefore, it can be considered that high stress plays a more important role in the initiation stage.

3. Development stage.

The development stage is the stage of continuous flow of coal particles and gas two-phase flow, often a relatively long and continuous process. After experiencing the intense energy release during the initiation stage, the stress inside the coal body is released, the force chain between particles also becomes longer, and the porosity inside the coal body also increases. The energy of coal particles is rapidly lost in the movement towards the outburst window and accumulates at the front end of the injection channel, thereby preventing the movement of gas and particles. The stress inside the channel is low and the particles are loose, causing gas desorption and resulting in high air pressure inside the channel. When the gas pressure is high enough, the gas breaks through the blocked particle pile and sprays out with coal particles. This process is constantly repeated, resulting in coal and gas outbursts exhibiting pulsating characteristics.

4. Termination stage.

The process of highlighting will not continue for a long time. With the release of gas in the coal body, when the combined force of gas pressure and stress in the pores is insufficient to break through the binding of particles, coal and gas outburst stops.

## Conclusion

1. The numerical model of particle flow for coal and gas outburst is in line with reality, and the extraction of various parameters is also relatively convenient. Especially, the simulation of coal and gas outburst processes is more intuitive compared to other numerical simulation methods, and can be applied in the related research of coal and gas outburst.
2. The concentrated stress caused by excavation of tunnels can reach 1.2 times the in-situ stress. High stress causes the adhesion between coal particles to break down and releases elastic properties into the outburst window, which is the main reason for the initiation of coal and gas outbursts.
3. The release of elastic energy leads to a decrease in stress inside the coal body, and the elongation of the force chain between particles leads to rapid desorption of gas. Coal particles block the gas release channel, causing coal and gas outbursts to pause until the amount of released gas is sufficient to break through the blocked particle collection and restart, resulting in pulsating characteristics of coal and gas outbursts.

## Data Availability

The datasets used during the current study available from the corresponding author on reasonable request.
